# Three-Dimensional Culture of Mouse Spermatogonial Stem Cells Using A Decellularised Testicular Scaffold

**DOI:** 10.22074/cellj.2020.6304

**Published:** 2019-07-31

**Authors:** Nasrin Majidi Gharenaz, Mansoureh Movahedin, Zohreh Mazaheri

**Affiliations:** 1 Department of Anatomical Sciences, Faculty of Medical Sciences, Tarbiat Modares University, Tehran, Iran; 2 Basic Medical Science Research Center, Histogenotech Company, Tehran, Iran

**Keywords:** Extracellular Matrix, Scaffold, Spermatogonial Cells, Testis

## Abstract

**Objective:**

Applications of biological scaffolds for regenerative medicine are increasing. Such scaffolds improve cell attachment, migration, proliferation and differentiation. In the current study decellularised mouse whole testis was used as a natural 3 dimensional (3D) scaffold for culturing spermatogonial stem cells.

**Materials and Methods:**

In this experimental study, adult mouse whole testes were decellularised using sodium dodecyl sulfate (SDS) and Triton X-100. The efficiency of decellularisation was determined by histology and DNA quantification. Masson’s trichrome staining, alcian blue staining, and immunohistochemistry (IHC) were done for validation of extracellular matrix (ECM) proteins. These scaffolds were recellularised through injection of mouse spermatogonial stem cells in to rete testis. Then, they were cultured for eight weeks. Recellularised scaffolds were assessed by histology, real-time polymerase chain reaction (PCR) and IHC.

**Results:**

Haematoxylin-eosin (H&E) staining showed that the cells were successfully removed by SDS and Triton X-100. DNA content analysis indicated that 98% of the DNA was removed from the testis. This confirmed that our decellularisation protocol was efficient. Masson’s trichrome and alcian blue staining respectively showed that glycosaminoglycans (GAGs) and collagen are preserved in the scaffolds. IHC analysis confirmed the preservation of fibronectin, collagen IV, and laminin. MTT assay indicated that the scaffolds were cell-compatible. Histological evaluation of recellularised scaffolds showed that injected cells were settled on the basement membrane of the seminiferous tubule. Analyses of gene expression using real-time PCR indicated that expression of the *Plzf* gene was unchanged over the time while expression of *Sycp3* gene was increased significantly (P=0.003) after eight weeks in culture, suggesting that the spermatogonial stem cells started meiosis. IHC confirmed that PLZF-positive cells (spermatogonial stem cells) and SYCP3-positive cells (spermatocytes) were present in seminiferous tubules.

**Conclusion:**

Spermatogonial stem cells could proliferate and differentiated in to spermatocytes after being injected in the decellularised testicular scaffolds.

## Introduction

The process of spermatogenesis is regulated by the endocrine system and testicular paracrine factors (1). In this process, germ cells are in contact with basement membrane and somatic cells that were located in seminiferous tubules. Hormonal and paracrine factors along with Sertoli cells and basement membrane are the main component of specialized microenvironment called a niche that promotes self-renewal of germ cells (2, 3). Impairment of each of these hormones and factors could lead to infertility.

In order to study the biology of spermatogonial stem cells and for a better-understanding of factors that regulate male fertility, *in vitro* culture techniques are commonly used (4). The chosen *in vitro* culture system for establishment of spermatogenesis should provide the right situation for communication between somatic and germ cells and the extracellular matrix (ECM). This could provide an environment similar to somniferous tubules of the testis (5). So, in order to mimic the local microenvironment for homing and attachment of germ and somatic cells, biological scaffolds and growth factors could be considered. These scaffolds have been successfully used for the regeneration of several organs including the lungs, pancreas, liver, and small veins (6, 7). Biological scaffolds are produced by decellularisation of actual tissues. In this process, the cells are removed from the tissues while the ECM components remain on what is referred to as a scaffold (8). These proteins provide structural and biochemical support for cell adhesion, proliferation, migration, and cell to cell interactions. Therefore, development of biological and biocompatible scaffolds could be beneficial for *in vitro* culture systems of germ cells. In recent years, applications of these scaffolds for *in vitro* spermatogenesis have been considered. Baert et al. (9) demonstrated natural testicular scaffold could support the self-assembly of human testicular cells to organoid structures. However, they reported that seeding testicular cells on decellularised scaffolds could not produce a testis with a typical cytoarchitecture. 

In another paper, Sertoli cells (10) were seeded on a testicular scaffold. Their results showed that the testicular scaffold could increase the proliferative activity of the Sertoli cells. They did not, however, investigate the spermatogonial stem cells differentiation in the presence of testicular scaffolds. In the present study mouse spermatogonial stem cells were injected in to whole testicular scaffolds via efferent ductuli, then cultured on agarose gels for evaluation of spermatogonial stem cells differentiation. 

## Materials and Methods

### Testes donors

In this experimental study, fifteen male Naval Medical Research Institute (NMRI) mice (8 weeks old) were used for the production of whole testicular scaffolds. The mice were in an animal house under controlled conditions (12 hour light/dark cycles). All animal procedures were conducted using guidelines approved by the Ethical Committee of Medical Sciences Faculty at the Tarbiat Modares University (Permission No. IR.TMU. REC.1394.269). 

### Organ harvest and decellularisation protocol 

Mice were euthanized using chloroform, then sacrificed by cervical dislocation. Subsequently, testes were removed from the mice. The capsules of the testes were perforated using an insulin syringe (29 gauge) and then washed with phosphate-buffered saline (PBS, Invitrogen, Switzerland) to remove residual blood. Decellularisation was done at 25°C using an orbital shaker (50 rpm). The washed testes were immersed in 0.5% (v/v) sodium dodecyl sulfate (SDS, Sigma, USA), then in 0.5% (v/v) Triton X-100 (Sigma, USA), both of which had been diluted in distilled water for 18 hours. Next, the scaffolds were washed extensively with PBS for 24 hours. Decellularised scaffolds were disinfected by 0.1% peracetic acid in 4% ethanol for 2 hours, and washed three times in sterile PBS for 4 hours each (11). 

### Scaffolds analyses

Fixation of the scaffolds was performed by incubation in 10% formalin solution in PBS at 25°C for 24-48 hours. The fixed scaffolds were then dehydrated by incubation in graded alcohol (each alcohol for 20 minutes). After embedding them in paraffin, they were cut into 5 µm-thick sections for histological evaluation. H<E (Sigma, USA) staining was performed on paraffin sections for evaluation of detergent efficacy to remove the cells and debris from the testes. Preservation of glycosaminoglycans (GAGs) and collagen in decellularised scaffolds were assessed by alcian blue (Sigma, USA) and Masson’s trichrome staining (Sigma, USA), respectively. Alcian blue was diluted 1:100 in hydrochloric acid (0.1 M). On these samples nuclear fast red (Sigma, USA) was used for counter-staining (12, 13). Also, preservation of ECM proteins, including fibronectin, collagen IV, and laminin in decellularised scaffolds was evaluated by immunohistochemistry (IHC). Initially, the sections were transferred to a 60°C oven for de-waxing, then further cleared in xylene. Later, they were rehydrated by alcohol gradient and washing in water. Then they were incubated in citrate (10 mM pH=6.0) for 20 min for antigen retrieval. Then the samples were permeabilized by triton X-100 for 40 minutes and incubated with anti-fibronectin (mouse monoclonal IgG, Elabscience Biotechnology Inc., USA), anti-collagen IV (mouse monoclonal IgG, Elabscience Biotechnology Inc., USA), and anti-laminin (rabbit polyclonal IgG, Abcam, USA). The secondary antibody was Alexa Fluor 488 (goat anti-mouse IgG, Invitrogen, USA) and Texas Red (Goat anti-rabbit IgG, Abcam, USA). Photomicrographs were taken with an Olympus microscope (Olympus, Center Valley, PA, USA). 

### Analysis of DNA content

DNA was isolated from 25 mg wet weight of intact and decellularised testes using a QIAamp DNA Mini Kit (Qiagen, Germany) (14). The concentration of DNA content was measured using a NanoDrop 2000 C UV-Vis spectrophotometer (Thermo Scientific, Venlo, Netherlands) at 260 nm. Each experiment was repeated five times.

### DAPI staining

Intact and decellularised testes were evaluated using 0.5 mg/mL blue-fluorescent 4, 6-diamidino-2-phenylindole (DAPI, Sigma, USA) for visualizing dsDNA. The DAPI solutions were diluted in PBS to 30 nM and were pipetted directly on each tissue section. They were kept in a dark room for 30 minutes. After washing with PBS, the slides were examined using an inverted fluorescence microscope (15). 

### Cytotoxicity assay 

Cytotoxicity of the scaffolds was evaluated by 3-[4, 5-dimethyl (thiazol-2yl)-3,5diphenyl] tetrazolium bromide (MTT, Sigma, USA) test, which assesses the viability of the cells. The scaffolds were cut into 2×2×2 mm^3^ fragments and placed in a 96-well plate. Mouse embryonic fibroblast (MEF) cells were isolated according to Jozefczuk’s protocol (16). Then, 3×10^4^ cells per well were seeded on the testicular scaffolds and cultivated in DMEM containing 10% fetal bovine serum (FBS, Gibco, Germany) for 72 hours. MTT assay was performed after 24 and 72 hours using the following protocol. Initially, 200 µL of medium containing MTT (0.5 mg/mL) was added to each well. Then they were incubated at 37°C for 4 hours for formazan formation. After removing the medium, the obtained formazan was dissolved in dimethyl sulfoxide (DMSO, Sigma, USA). The optical density (OD) of the supernatants was measured using a microplate reader (Beckman, Fullerton, CA) at 570 nm. Five replicates were performed for each sample (17).

### Recellularization of testicular scaffolds

#### Isolation and culture of spermatogonial stem cells 

After euthanizing 5 male NMRI mouse pups (6 days old), their testes were removed and placed immediately in a 3.5-cm dish containing PBS and were cooled on ice. Spermatogonial stem cells were isolated according to the protocol described by Mirzapour et al (18) and subjected to a two-step enzymatic digestion with 0.5 mg/ml trypsin, 0.5 mg/ml collagenase IV and 0.5 mg/ml hyaluronidase (all from Sigma, USA). For cell viability assay, a sample of the cells was mixed with trypan blue and transferred to a hemocytometer, where the live unstained cells were counted under a light microscope. Following the enzymatic digestion step, the cell suspension was cultivated in alpha minimum essential medium (αMEM, Bio-Ideal, Iran) supplemented with 10% FBS at 34°C in 5% CO_2_ for two weeks. 

### Identification of spermatogonial stem cells 

The identity of the isolated spermatogonial stem cells was verified by tracing the PLZF protein (19) in the obtained colonies from the cell suspension after two weeks in culture. Fixed cells were incubated overnight with a mouse monoclonal anti-PLZF antibody (mouse monoclonal IgG, sc-28319 Santa Cruz Biotechnology, USA, diluted 1:100) at 37°C. Following PBS washes they were incubated with an Alexa 488-conjugated secondary antibody (goat anti-mouse IgG, USA, diluted 1:200 in PBS) for 1 hour in the dark at 25°C. Nuclei were stained by propidium iodide (PI). 

### In vitro transplantation of spermatogonial stem cells in to whole testicular scaffolds 

Initially, the cell suspension was stained with trypan blue, then 10 µl of the stained cells were injected by a glass needle into the end of the efferent ductuli and the opening of the rete decellularised testes. Then recellularized testicular scaffolds were cut into 1×1×1 mm pieces under a stereomicroscope and cultured on agarose gel. An agarose support layer and a culture medium with specific compositions and growth factors were prepared according to the protocol by Yokonishi and colleagues (20). The culture medium supplemented with 10% knockout serum replacement (KSR, USA), 60 ng/ ml progesterone (Invitrogen, UK), 30 ng/ml beta-estradiol (Pepro Tech, USA), 20 ng/ml epithelial growth factor (EGF, Pepro Tech, USA), 10 ng/ml basic fibroblast growth factor (bFGF, Pepro Tech, USA), and 10 ng/ml leukemia inhibitory factor (LIF, Royan, Iran). Pieces of the recellularized scaffolds were placed gently in the middle of the agarose layer to prevent them from floating. They were cultivated under static conditions at 37°C with 5% CO_2_ for up to 8 weeks. Cell-free testicular scaffolds were cultured under the same conditions as the control. The culture medium was replaced with fresh medium twice a week. The samples (20 pieces) were collected for histological and molecular evaluation at the end of the second and eighth weeks of culturing. 

### Histology and immunohistochemistry

Recellularized testicular scaffolds and intact testes as the positive control group were fixed in 10% formalin solution in PBS at 25°C for 24-48 hours. Then samples were dehydrated by graded alcohol. After embedding in paraffin, they were cut into 5 µm-thick sections for histological evaluation. H<E staining was performed on samples cultured for two and eight weeks. For IHC, primary antibody PLZF (mouse monoclonal IgG, sc-28319 Santa Cruz Biotechnology, USA, diluted 1:100) and SYCP3 (mouse monoclonal IgG, Santa Cruz Biotechnology, USA, diluted 1:100) were used. The secondary antibody was Alexa Fluor 488 (goat anti-mouse IgG, Invitrogen, USA) and the nuclear stain DAPI (Life Technologies, USA) was used for counterstaining. Photomicrographs were taken with an Olympus Microscope (Olympus, Center Valley, PA). 

### Real-time polymerase chain reaction studies for analysis of gene expression

The expression of *Plzf* and *Sycp3* genes were assessed by real-time PCR. For extraction of total RNA from samples, RNX-Plus™ KIT (Cinna Gen, Iran) was used, then RNA was treated with DNase I (Fermentase, USA) to remove the genomic contamination. The RNA concentrations were measured by a biophotometer (Eppendorf, USA). cDNA was synthesized from 1000 ng RNA using a cDNA kit (Fermentase, Germany) (21). Primers for *Plzf* and *Sycp3* genes were designed using the NCBI website and were synthesized by Cinna Gen (Iran, Table 1). The PCR reactions were done using Master Mix and SYBR Green (Fluka, Switzerland) in a StepOne™ thermal cycler (Applied Biosystems, USA). Melting curve analyses were used for confirmation of the quality of the PCR reactions. A standard curve was used to determine the efficiency of each gene (logarithmic dilution of cDNA from the samples). In addition, this process was repeated in triplicates for all the target and reference (*ß-actin*) genes. The target genes were normalized to the reference gene. 

**Table 1:** Primer sequences for real time- polymerase chain reaction


Gene	Primer sequence (5ˊ-3ˊ)	Accession number	Product length

*β-actin*	F:TTACTGAGCTGCGTTTTACAC	NM_007393.5	90
		R:ACAAAGCCATGCCAATGTTG	
*Plzf*	F:GCTGCTGTCTCTGTGATGG	NM_001033324.3	153
		R:GGGCTGATGGAACATAGGGG	
*Sycp3*	F:TCAGCAGAGAGCTTGGTCGG	NM_011517.21	118
		R:GATGTTTGCTCAGCGGCTCC	


### Statistical analysis

All data are presented as mean values ± standard error. SPSS software (version 16.0, Chicago, USA) was used for data analysis. DNA content and MTT data analysis were conducted using an independent sample t test. Real-time PCR data analysis was performed by one-way analysis of variance (ANOVA) followed by Tukey’s post hoc test. Three replicates were done per sample. P=0.05 was considered statistically significant. 

## Results

### Characterization of decellularised testicular scaffolds 

**Fig.1:**
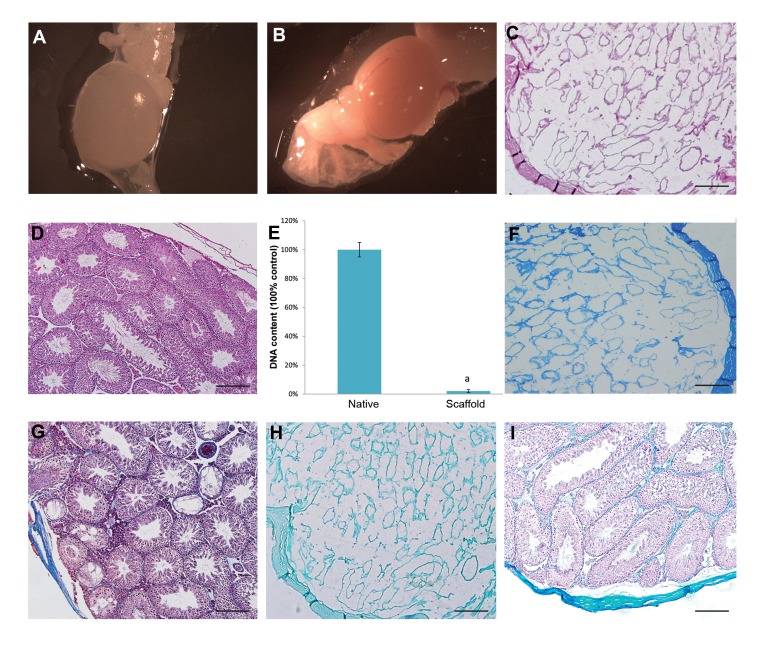
Characterization of decellularised testes. **A.** Macroscopic images showed that decellularised testes were completely translucent while, **B.** Intact testes were opaque, **C.** Histological comparison of decellularised, **D.** Intact testes by H&E staining exhibited the elimination of the cells, **E.** DNA quantification confirmed removal of 98% of the DNA from the tissue. a; Indicated significant difference with intact testis, **F.** Masson’s trichrome staining showed collagen preservation in decellularised, **G.** Intact testes, **H.** Alcian blue staining confirmed glycosaminoglycans (GAGs) retention in decellularised, and I. Intact tests (scale bar: 100 µm).

**Fig.2:**
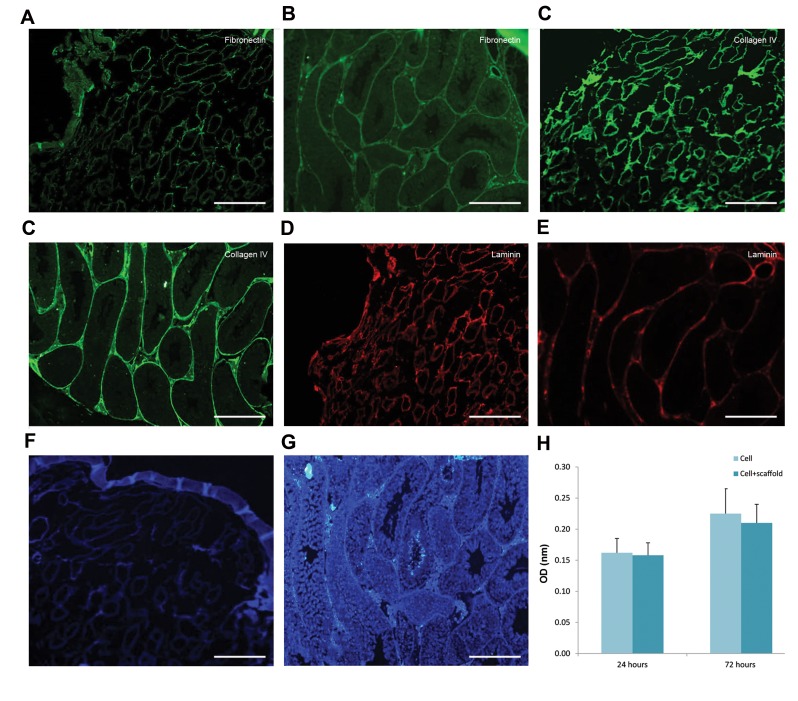
Protein and nucleic acid analyses of the decellularised scaffolds and intact testes. **A.** Representative images of fibronectin expression in decellularised scaffolds, **B.** Intact testis, **C.** Collagen IV expression in decellularised scaffolds, **D.** Intact testis, **E.** Laminin expression in decellularised scaffolds, **F.** Intact testis, **G.** DAPI staining of decellularised scaffolds, **H.** Intact testis, and **I.** Evaluation of scaffold cytocompatibility using MTT test did not show any significant difference in the optical density (OD) values, meaning that the cells proliferated at a rate similar to that of the controls (scale bar: 100 µm).

Macroscopically decellularised testes, which retained the gross shape of the whole organ, were completely translucent (Fig .1A), while intact testes were opaque (Fig .1B). Histological evaluation by H&E staining showed that the cells were removed by SDS and Triton X-100 (Fig .1C). Intact testes were stained as control (Fig .1D). In order to evaluate the efficiency of the decellularisation protocol more accurately, DNA content was measured as well. Analysis of DNA content indicated that approximately 98% of the DNA was successfully removed from the testes. This further confirmed that our decellularisation protocol was efficient (Fig .1E). Masson’s trichrome staining showed blue stained collagen fibers in the decellularised testes, while no red stained areas, which would indicate cell residues, were observed (Fig .1F). Intact testes were stained as control (Fig .1G). The maintenance of GAGs in scaffolds was assessed by alcian blue staining, which demonstrated that GAGs were in fact preserved (Fig .1H). Intact testes were stained as control (Fig .1I). IHC staining verified the preservation of fibronectin (Fig .2A, B), collagen IV (Fig .2C, D), and laminin (Fig .2E, F) in the decellularised testes and intact testes respectively, with no detectable DAPI staining (Fig .2G) in the decellularised testes. Intact testes were stained as control (Fig .2H). These findings suggest that cellular elements were eliminated completely while ECM proteins including fibronectin, Collagen IV, and laminin have remained.

### Recellularization of decellularised testicular scaffolds following *in vitro* transplantation

**Fig.3:**
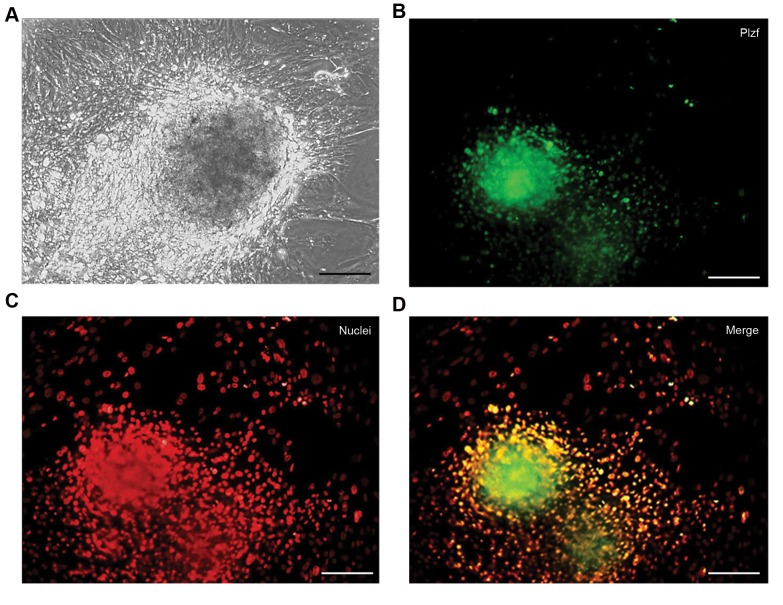
Characterization of spermatogonial stem cells harvested from neonatal mouse testes. **A.** Phase contrast images of spermatogonial stem cell colonies after two weeks of culture, and **B-D.** IHC staining of spermatogonial stem cell colonies with PLZF marker. Cell nuclei were stained by propidium iodide (PI) (scale bar: 30 µm).

**Fig.4:**
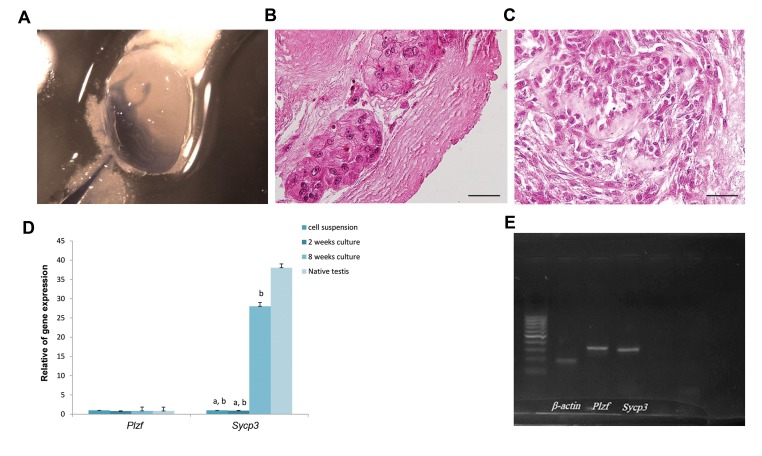
Characterization of cell injected scaffolds. **A.** Gross image of repopulated testicular scaffolds using in vitro transplantation (IVT) of spermatogonial stem cells, **B.** Haematoxylin-eosin images of the recellularized scaffolds after two weeks (scale bar: 20 µm), **C.** Eight weeks of culturing. Representative image of decellularised scaffolds without IVT after eight weeks in culture (scale bar: 20 µm), **D.** Relative gene expression of recellularized scaffolds after two and eight weeks of culture, and **E.** Bands of *Plzf* and *Sycp3* genes, and *ß-actin* gene as the housekeeping control were obtained by real-time polymerase chain reaction (PCR). a; Indicated significant difference with samples cultured for eight weeks and b; Indicated significant difference with intact testis.

To evaluate the potentials of decellularised testicular tissue as a scaffold for tissue engineering, it was recellularized using *in vitro* transplantation (IVT) of murine spermatogonial stem cells. Initially, to determine the cytotoxicity of the scaffold, MTT testing was performed. The result of the MTT assay showed that decellularised testicular scaffolds had no detectable effects on the MEF proliferative activity after 24 and 72 hours of culture (Fig .2I). Spermatogonial colonies were obtained after two weeks culture of testicular cell suspension (Fig .3A). PLZF protein was expressed in these colonies (Fig .3B-D). After IVT of spermatogonial stem cells, which mixed with trypan blue was completed, the cell suspension was spread in the seminiferous tubules, and approximately 20 to 40% of the decellularised testis was filled (Fig .4A). Histological examination of recellularized scaffolds was conducted after two and eight weeks of culture. H&E staining showed that injected spermatogonial stem cells resided on the basement membrane of the seminiferous tubules and interstitium after two weeks of culture (Fig .4B). Organoid like structures was seen after eight weeks of culture (Fig .4C). 

**Fig.5:**
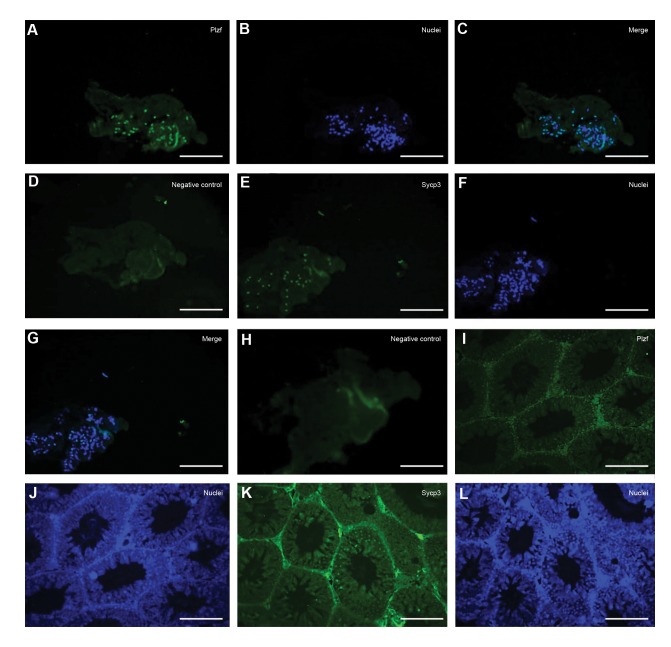
Immunohistochemistry (IHC) images of the cell-injected scaffolds and intact testes. **A-C.** IHC staining showed PLZF-positive cells in scaffolds cultured for eight weeks, **D.** Negative control of PLZF, **E-G.** SYCP3-positive cells in scaffolds cultured for eight weeks, **H.** Negative control of SYCP3, I, J. Positive control of PLZF, **K,** and **L.** SYCP3 in adult testis (scale bar: 50 µm).

In order to evaluate the expression of spermatogenesisspecific genes, real-time PCR was performed. Our results indicated that *Plzf* gene expression did not show any significant difference between samples cultured for two and eight weeks, while expression of *Sycp3* genes significantly increased (P=0.003). Also, expression of *Sycp3* gene in samples cultured for two and eight weeks was significantly lower compared to intact testes (P=0.003, Fig .4D). Bands of *Plzf* and *Sycp3*, and *ß-actin* genes were detected on gel electrophoresis (Fig .4E). Detection of germ cell markers at the protein level was confirmed via immunostaining of recellularized scaffolds. IHC confirmed PLZF-positive cells (Fig .5A-C) were present in the recellularized scaffolds after eight weeks of culturing. The scaffolds without cell injection didn’t expressed the PLZF protein (Fig .5D). SYCP3-positive cells (Fig .5E-G), were present in the recellularized scaffolds after eight weeks of culturing. The scaffolds without cell injection didn’t expressed the SYCP3 protein (Fig .5H). Mouse adult testis was stained as a positive control for PLZF (Fig .5I, J) and SYCP3 (Fig .5K, L) markers. 

## Discussion

Applications of ECM scaffolds are increasing for the establishment of artificial organ structures in order to mimic organ functions (22). This study investigated the use of decellularised whole testicular scaffold to support proliferation and differentiation of spermatogonial stem cells *in vitro*. Initially, murine whole testes were decellularised using SDS and Triton X-100. DNA content analyses demonstrated 98% cell removal, suggesting that our decellularisation method efficiently removes testicular cellular components. Our results were in line with other studies on SDS plus Triton X-100 application for tissue decellularisation in tendon-bone, small-diameter blood vessels and pericardium and cardiac tissues (13, 23-25). Preservation of ECM proteins is necessary in tissue engineering in order to facilitate interactions between cell and matrix (26). Main components of testicular ECM are laminin, fibronectin, and collagens that were detected in testicular scaffolds using IHC. Baert et al. have reported that decellularisation of human testes by detergents could preserve the components of basement membrane including collagens, laminin, and fibronectin (12). Collagens are necessary for the maintenance of tissues structure, laminin is an important adhesion molecule, and fibronectin supports cell attachment and migration (27). So, these proteins are important factors for successful attachment of spermatogonial stem cells to the basement membrane of the seminiferous tubules (10). Cytotoxicity assay by MTT showed that decellularised testicular scaffolds had no harmful effects on MEF proliferative activity. The cells metabolized the MTT substrate, indicating that MEF cell mitochondria were functional on decellularised testicular scaffolds, which in turn resulted in a good overall cell viability and proliferation. Thus, the decellularised testicular scaffolds were confirmed to be cell-compatible. 

Subsequently, these scaffolds were recellularized by injection of spermatogonial stem cells via efferent ductuli to whole testicular scaffolds and were cultured on agarose gel for eight weeks in order to evaluate the differentiating potentials of spermatogonial stem cells. In the previous studies (9, 10) the cell suspension was seeded directly onto scaffolds, while in our study the cells were injected to rete testes and seminiferous tubules for facilitating attachment of the spermatogonial stem cells to the basal lamina, their colonization and differentiation. H&E staining showed that the injected cells resided on the basement membrane of the seminiferous tubule and interstitium after two weeks of culture. Organoid- like structures were seen in the samples cultured for eight- weeks. Baert et al. (9) reported natural testicular scaffolds could support the self-assembly of human testicular cells to organoid structures. So, injection of the cells into seminiferous tubules or seeding the cell on to the scaffolds results in development of a similar structure. In decellularised scaffolds without IVT, seminiferous tubules collapsed and no cells were seen on the scaffolds after eight weeks of culture. Injection of the cells to the seminiferous tubules resulted in cell proliferation and of secretion of ECM proteins. In another study in 2018, Vermeulen, et al. declared that seeding Sertoli cells onto testicular scaffolds could rise the proliferative activity of the Sertoli cells (10). They did not investigate the fate of spermatogonial stem cells in the presence of the scaffolds. 

For identification of the nature of the observed cells in seminiferous tubules, cell-specific gene expression was evaluated over time. The expression of Plzf gene did not show any significant differences between two and eight weeks cultured samples. *PLZF* is a pluripotency marker that plays an important role in proliferation and self- renewal of spermatogonial stem cells (28). Baert et al. (29) reported that key markers of human spermatogonial stem cells, such as *Plzf, Uchl1,* and *Thy1*, were easily detected in the mRNA samples from spermatogonial stem cells, which had been cultured on testicular scaffolds. Pendergraft et al. (30) reported that *Plzf* expression remained unchanged in testicular organoid during the culture period. This could indicate that the spermatogonial stem cells pool in a scaffold is able to maintain the undifferentiated state for eight weeks in culture. Since differentiation of spermatogonial stem cells is a key aspect of normal spermatogenesis, we further evaluated *Sycp3* gene expression. The results showed a significant increase in samples that had been cultured for eight weeks compared to those cultured for two weeks. SYCP3 is a meiotic marker that elaborates in recombination and separation of chromosomes in meiotic division (31). Deletion of SYCP3 in mice causes problems in fertility. Also, lack of SYCP3 in males could induce apoptosis in spermatocytes and may prevent formation of synaptonemal complexes. Aarabi et al. (32) showed that the expression level of testicular sycp3 mRNA is correlated with the degree of spermatogenic failure. The expression of SYCP3 was not seen in patients with testicular atrophy, Sertoli cell- only syndrome, or arrest of spermatogonial stem cells. In the current study, spermatogonial stem cells could proliferate and initiate meiosis, but spermatocytes did not complete spermiogenesis to produce functional sperms. 

In addition to transcripts level, immunostaining of samples confirmed the presence of spermatogonial stem cells expressing PLZF and spermatocyte cells expressing SYCP3 proteins in samples cultured for eight weeks. Taken together, these data indicate that our scaffold has the capacity to support spermatogonial stem cells attachment and differentiation through the spermatocyte formation stage. We could not find round spermatid or spermatozoa after eight weeks of culturing. This may be due to the cultivation system and the types of culture medium supplements. In the present study, the culture media were supplemented by several factors including LIF, BFGF, EGF, estradiol, progesterone, and glial cell line-derived neurotrophic factor (GDNF) to improve proliferation of spermatogonial stem cells and to induce their differentiation. From these factors, LIF, BFGF, and estradiol induce proliferation and lead to survival of spermatogonial stem cells in culture (33, 34). EGF activates differentiation of germ cells, but reduces the proliferation rate of spermatogonial stem cell (35). Progestin stimulates early stages of spermatogenesis (36). GDNF has an important role in self-renewal and differentiation of germ cells (37). It seems that our supplemented medium with a verity of factors with different effects on proliferation and differentiation may have impaired the spermatogenesis process. Therefore, further studies should be conducted to focus on improving the culture system and culture medium. This could possibly be done by using a dynamic culture system or hydrogel developed from decellularised testicular ECM. Recently, growth factors have been successfully conjugated to biological or synthetic scaffolds. The cells that have interactions with the matrix could use these conjugated factors, so that they provide extremely localized signals to regulate the cell fate (38). Applications of growth factors conjugated to decellularised testicular scaffolds for induction of differentiation in spermatogonial stem cells could be considered in future studies. 

## Conclusion

Our decellularised testicular scaffolds were cell- compatible and did not have a harmful effect on MEF and spermatogonial stem cells viability. Recellularization of this scaffold using the IVT method could help spermatogonial stem cells to differentiate to produce the spermatocytes. 
